# NIA Division of Neuroscience

**DOI:** 10.1093/geroni/igab046.1050

**Published:** 2021-12-17

**Authors:** Eliezer Masliah

**Affiliations:** National Institute on Aging, Bethesda, Maryland, United States

## Abstract

Dr. Masliah will discuss research priorities for the Division of Neuroscience. Dr. Masliah will also be available for small group discussion.

